# The Role of MicroRNA in the Modulation of the Melanocortinergic System

**DOI:** 10.3389/fnins.2017.00181

**Published:** 2017-04-05

**Authors:** Adel Derghal, Mehdi Djelloul, Jérôme Trouslard, Lourdes Mounien

**Affiliations:** ^1^Physiologie et Physiopathologie du Système Nerveux Somatomoteur et Neurovégétatif (PPSN), Aix Marseille UniversityMarseille, France; ^2^Department of Cell and Molecular Biology, Karolinska InstituteStockholm, Sweden

**Keywords:** microRNA, melanocortin, feeding behavior, hypothalamus, energy homeostasis

## Abstract

The central control of energy balance involves a highly regulated neuronal network within the hypothalamus and the dorsal vagal complex. In these structures, pro-opiomelanocortin (POMC) neurons are known to reduce meal size and to increase energy expenditure. In addition, leptin, a peripheral signal that relays information regarding body fat content, modulates the activity of melanocortin pathway neurons including POMC-, Agouti-related peptide (AgRP)/Neuropeptide Y (NPY)-, melanocortin receptors (MC3R and MC4R)-expressing neurons. MicroRNAs (miRNAs) are short non-coding RNAs of 22–26 nucleotides that post-transcriptionally interfere with target gene expression by binding to their mRNAs. Evidence has demonstrated that miRNAs play important roles in the central regulation of energy balance. In this context, different studies identified miRNAs including miR-200 family, miR-103, or miR-488 that could target the genes of melanocortin pathway. More precisely, these different miRNAs can modulate energy homeostasis by affecting leptin transduction pathway in the POMC, or AgRP/NPY neurons. This article reviews the role of identified miRNAs in the modulation of melanocortin pathway in the context of energy homeostasis.

## Introduction

Overweight and obesity are significant risk factors for various chronic diseases, including cancer, heart diseases, and type 2 diabetes. In 2014, World Health Organization estimated that more than 1.9 billion adults were overweight. Of these over 600 million were obese. Dramatically, 41 million children under the age of 5 were overweight or obese in 2014. With such a high and expanding prevalence, and considering the associated diseases, obesity has an important economic impact on health care systems. For instance, the global medical costs related to obesity were estimated to reach up to 147 billion dollars per year in the USA (Bariohay et al., 2011). The direct health care costs linked to obesity in industrialized country can exceed 7% of the total health care costs (Bariohay et al., 2011). Environmental factors lead to an increase in the proportion of obese people. To date, the main treatment against obesity is to decrease caloric intake combined with an increase in the physical activity. The major limit of this treatment is the low achievement rate in the long haul, revealing the need for additional medical approaches. Then, given the expanding number of obese patients, obesity research is critical in the medication improvement field.

**Figure 1:**
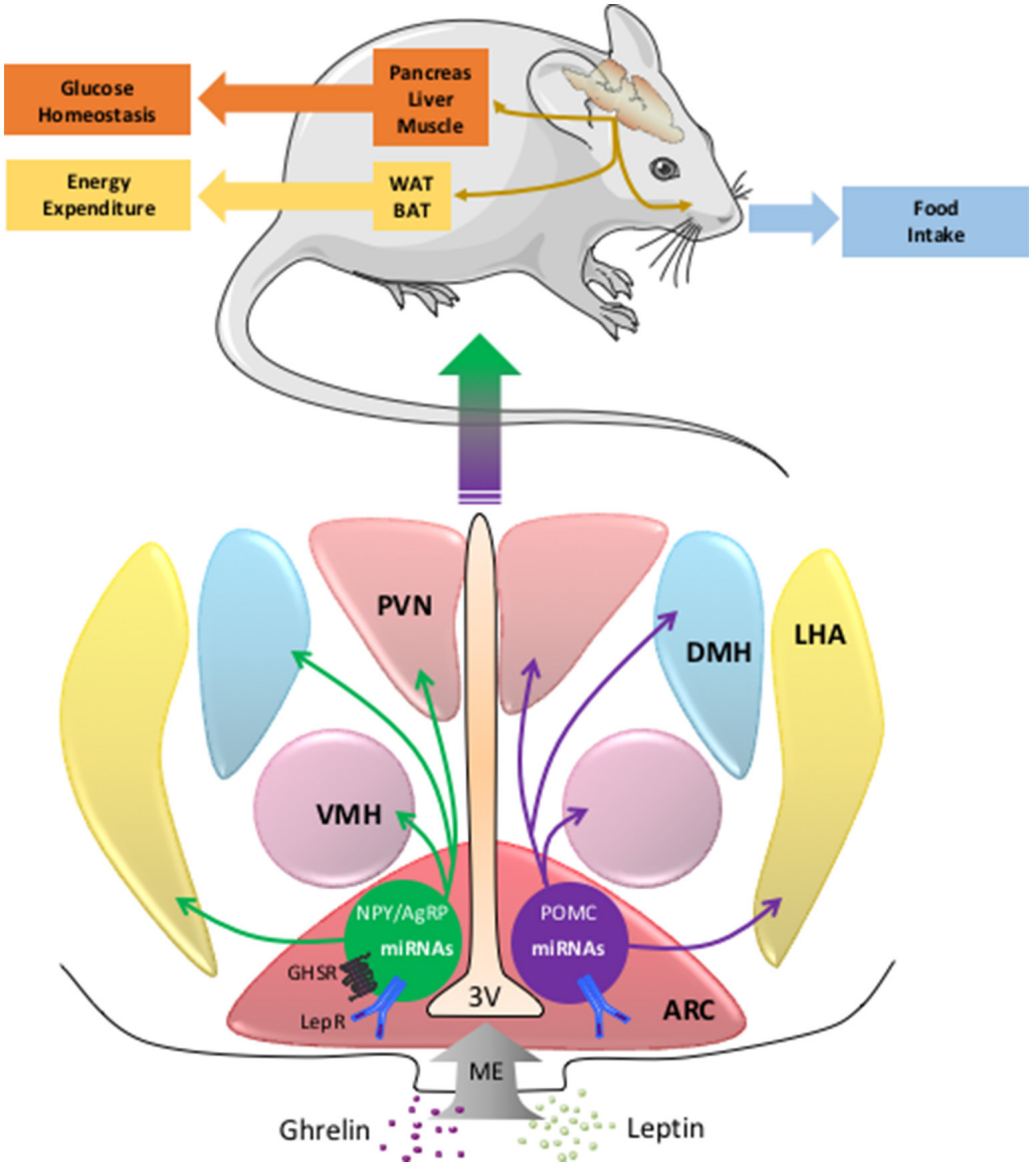
**The miRNAs in POMC and NPY/AgRP neurons and the central regulation of energy balance**. The neurons of the melanocortin pathway integrate peripheral signals as ghrelin or leptin that modulate the expression of POMC, NPY, AgRP, and miRNAs genes. Then the melancortin pathway elicits adaptive responses like food intake, energy expenditure, or glucose homeostasis. AgRP, agouti related peptide; ARC, arcuate nucleus; BAT, brown adipose tissue; DMH, dorsomedial hypothalamus; LepRb, leptin receptor type b; LHA, lateral hypothalamus area; ME, median eminence; miRNA, microRNA; NPY, neuropeptide Y; GHSR, ghrelin receptor; POMC, pro-opiomelanocortin; PVN, paraventricular nucleus; VMH, ventromedial hypothalamus; WAT, white adipose tissue; 3V, third ventricle.

The control of energy homeostasis involves endocrine and neuronal mechanisms that modulate the balance between caloric absorption and energy expenditure. In this context, the central nervous system (CNS) continuously follows modifications in metabolic parameters (i.e., glycemia or free fatty acids levels) or hormones (insulin, leptin, ghrelin, PYY3-36, GLP-1, and cholecystokinin) and elicits adaptive responses like food intake regulation or autonomic nervous system modulation of glucose homeostasis and energy expenditure (Figure 1). Among the brain regions involved in this regulation, the hypothalamus, and the dorsal vagal complex (DVC) in the brainstem play a pivotal role through specific neuronal networks (Berthoud, 2002, 2004; Morton et al., 2005, 2006; Schneeberger et al., 2014). More particularly, within the arcuate nucleus (ARC) of hypothalamus and the nucleus of the solitary tract (NTS) of DVC, pro-opiomelanocortin (POMC) neurons are important regulators of energy, and glucose homeostasis (Morton et al., 2006). In this context, leptin is an adipose-derived hormone that is crucial to maintain both normal body weight and insulin sensitivity by action in the hypothalamus (Balthasar et al., 2004; Coppari et al., 2005; Dhillon et al., 2006; Morton et al., 2006; van de Wall et al., 2008). This peripheral signal is detected by hypothalamic arcuate neurons expressing the anorexigenic peptide POMC or the orexigenic peptides Neuropeptide Y (NPY)/Agouti-related peptide (AgRP). These neurons project to melanocortin 3 and 4 receptor-expressing neurons located in hypothalamus and other brain structures (Morton et al., 2006). Together these neurons are called the melanocortin pathway and regulate feeding behavior, energy expenditure, and glucose homeostasis through activation of the autonomic nervous system and higher brain structures (Berthoud, 2002; Morton et al., 2006) (Figure 1).

One important goal of present research is to identify the molecular mechanism and the intracellular mediators allowing these POMC and NPY/AgRP neurons to respond to energy status variations. Then, it appears crucial to increase our knowledge of the mechanisms controlling the melanocortin system activity, particularly by the discovery of new signaling pathways involved in the control of POMC and AgRP genes expression by leptin. Such pathways should provide beneficial pharmacological targets, and lead to the development of new generation drugs that can safely and effectively treat overweight and obesity linked to leptin resistance. In this context, it has been recently discovered new mechanisms involved in the control of the expression of the melanocortin pathway's genes. In particular, epigenetic mechanisms, including DNA methylation, the modifications of histones, and specific microRNAs (miRNAs) expression, have been proposed to mediate the expression of the melanocortin system (Stevens et al., 2010, 2011; Funato et al., 2011; Cansell and Luquet, 2012; Schneeberger et al., 2015).

This review provides an insight into the new mechanisms of the regulation of the POMC, NPY, and AgRP genes and a focus on the function of the miRNAs in this process will be developed.

## The melanocortin system and regulation of energy homeostasis

As mentioned above, POMC-expressing neurons moderate food intake, glucose homeostasis, and energy expenditure (Cowley et al., 2001; Parton et al., 2007; Mounien et al., 2009, 2010). The prohormone POMC is cleaved into α-melanocyte-stimulating hormone (α-MSH) that binds to the melanocortin 3 and 4 receptors (MC3R and MC4R) on neurons located in the nucleus of the hypothalamus as well as in the DVC (Cummings and Schwartz, 2000; Jégou et al., 2003; Coll et al., 2004; Rossi et al., 2011). The activation of MC4R induced a decrease of the food intake and an increase of the energy expenditure and this receptor is also involved in glucose homeostasis. MC4R agonists provide therefore a potential tool for the treatment of metabolic disorders as obesity (Rossi et al., 2011; Zechner et al., 2013). Conversely, mutations in the POMC, MC3R, or MC4R genes cause common or massive early-onset obesity in humans, further supporting a crucial role for the melanocortin pathway in energy homeostasis (Krude et al., 1998; Farooqi and O'Rahilly, 2000; Lee, 2009). It is important to notice that AgRP has been described as an endogenous antagonist or inverse agonist of the melanocortin receptors (Cone et al., 1996; Ollmann et al., 1997). Altogether, these neurons belong to the central melanocortin system, a family of diverse cells that comprise POMC-, AgRP-, MC3R-, and MC4R-expressing neurons. These neurons regulate peripheral metabolism through the activation of the autonomic nervous system and higher brain structures to control energy homeostasis but also the arousal and reward systems (Berthoud, 2002; Morton et al., 2006). Recently, by using optogenetic approach, Aponte et al. found that POMC and AgRP neurons have counter-regulatory roles on the regulation of food intake, confirming the pivotal role of these neurons in the control of feeding behavior (Aponte et al., 2011). Regarding the action of leptin on this melanocortin pathway, deletion of SOCS-3, a negative regulator of the action of this hormone, in POMC neurons, improved glucose homeostasis and insulin sensitivity as well as resistance to high fat diet (HFD) (Kievit et al., 2006). Lately, the simultaneous disruption of insulin and leptin receptors induced insulin resistance in mice (Hill et al., 2010). Altogether, these data showed the main role of POMC neurons in the integration of peripheral signals, as leptin, reflecting the energy status of organism. In addition, leptin is required for the accurate development of the POMC neurons (Bouret et al., 2004; MacKay and Abizaid, 2014).

In addition to the communication between brain and peripheral organs, intracellular metabolic-sensing mechanisms in CNS neurons are also crucial for the control of the energy balance. For instance, it has been established that AMP-activated protein kinase (AMPK), the mammalian target of rapamycin (mTOR), and SIRT1 deacetylase in the hypothalamus, are essential for leptin sensing and then energy homeostasis. More precisely, inactivation of AMPK in POMC neurons induced obesity while SIRT1 in POMC neurons is required for adaptations against diet-induced obesity (Claret et al., 2007; Ramadori et al., 2010). In addition, it has also been demonstrated that epigenetic mechanisms such as histone modifications or DNA methylation are acknowledged to modulate POMC gene activity under different nutritional status (Stevens et al., 2010; Funato et al., 2011). Then, these data established that POMC gene expression is highly and tightly controlled by different mechanisms in order to regulate energy homeostasis by the modulation of appetite and energy expenditure.

## The microRNA and the melanocortin system

Gene expression can be controlled at the transcriptional or post-transcriptional levels as well as during and after the translation. In this context, it has recently been highlighted that small RNAs, miRNAs, play predominantly inhibitory regulatory roles by binding to the 3′ untranslated region (3'UTR) of message encoding RNAs. The miRNAs are small non-coding RNA molecules of 21 to 26 nucleotides that regulate gene expression (Bartel, 2004; Derghal et al., 2016). They were first discovered in *Caenorhabditis elegans* in 1993 and, later on, in vertebrates and plants (Lee et al., 1993; Wightman et al., 1993). These non-coding RNAs induced specific gene silencing by base pairing to 3'UTR of target messenger mRNAs. miRNAs exert their actions by inhibiting translation and by affecting mRNA stability and degradation (Bartel, 2004; Guo et al., 2010; Derghal et al., 2016). Based on computational algorithms, around 60% of human transcripts contain potential miRNA-binding sites within their 3′UTRs (Friedman et al., 2009). A single miRNA can potentially bind to more than 100 target mRNAs, and multiple miRNAs can cooperate to finely tune the expression of the same transcript (Doench and Sharp, 2004; Grimson et al., 2007; Selbach et al., 2008). The miRNAs play key roles in numerous physiological processes including cell proliferation, apoptosis, neurodevelopment, and tissue differentiation but also in pathological processes as cancer (Bartel, 2004). Interestingly, defects in miRNA biogenesis and function have been shown to contribute to the development of metabolic disorders. For instance, mir-14, mir-278, and let-7 are involved in the metabolism of lipid and glucose respectively (Krützfeldt and Stoffel, 2006; Frost and Olson, 2011).

As indicated before, miRNAs are important for neurodevelopment but also neurotransmission or synaptic plasticity (Díaz et al., 2014). In the case of the hypothalamus, several studies demonstrated that the miRNA transcriptome is different at different stages of development. For instance, Zhang et al. showed that 30 miRNAs including miR-7 and miR-191 are differentially expressed in the hypothalamus of the pig between stages P60, P120, and/or P180 (Zhang et al., 2013). More recently, a nice work showed robust changes in the expression of numerous miRNAs during the period of functional organization of the ARC and median eminence between stages P8–P14 and stages P21–P28 (Doubi-Kadmiri et al., 2016).

As mentioned above, hypothalamus and DVC are important for the detection of circulating nutrients and hormones and in turn, these neuronal structures modulate the pancreas, liver, and adipose tissue physiology through efferent pathways. The function of miRNAs in the hypothalamus and DVC has not been clearly addressed. However, as in the other organs involved in energy homeostasis, miRNAs undoubtedly play a key role in hypothalamus and DVC neurons, and particularly in the function of melanocortin pathway. In accordance with this point, it has been shown that in the anorexia mouse model, *anx/anx*, there is an alteration of miRNA machinery expression. In particular, an up-regulation of RISC genes (Dgcr8, Ago2, Fmr1, Ddx6, and Pabpc1) has been observed in the hypothalamus of *anx/anx* mice (Mercader et al., 2012). However, the link between the phenotype of the *anx/anx* mice (anorexia, hyperactivity, and ataxia) and the differential regulation of RISC genes need to be clarified.

A large number of miRNAs are expressed in the brain, and deletion of Dicer, a specific enzyme involved in miRNA maturation, in specific brain structures or neuronal cell type can lead to behavioral defect and neurodegeneration (Schaefer et al., 2007; Cuellar et al., 2008; Olsen et al., 2009; Hébert et al., 2010; Tao et al., 2011). Recently, it has been shown that Dicer is essential for the central control of energy homeostasis. In fact, the neuron-specific deletion of Dicer induced obesity in mice (Mang et al., 2015). Interestingly, brain transcriptome analyses in this obese mice model identified several obesity-related pathways as leptin signaling (Mang et al., 2015). In the hypothalamus, deletion of Dicer in the ARC of adult mice induced hyperphagia and obesity (Vinnikov et al., 2014). The group of Dr Claret also showed that the hypothalamic expression of Dicer is modulated by fasting (Schneeberger et al., 2012). In contrast, the expression of Dicer is increased in diet-induced obesity model and *ob/ob* mice (Schneeberger et al., 2012). Altogether, these results suggest that the expression of Dicer is modulated by nutrient availability. Interestingly, Dicer is expressed in 94% of POMC and NPY/AgRP neurons suggesting an important function of Dicer and Dicer-derived miRNA in the modulation of the POMC, AgRP, and NPY genes expression (Schneeberger et al., 2012).

It has been established that each tissue exhibit a specific profile of miRNA expression (Babak et al., 2004; Lee et al., 2008). First studies revealed an enrichment of several miRNAs including let-7c, miR-7a, miR-7b, miR-124a, miR-125a, miR-136, miR-138, miR-212, miR-338, and miR-451 in the hypothalamus of rodents (Farh et al., 2005; Bak et al., 2008). These observations have been confirmed in ARC and paraventricular (PVN) nucleus of the hypothalamus by illumina sequencing technology (Amar et al., 2012). And in particular, expression was high or moderate for about 20 miRNAs as let-7, miR-7a and b that may be used to define a common ARC/PVN profile of male Wistar rats (Amar et al., 2012). In the line of this observation, it has been demonstrated that miR-7a is expressed preferentially in NPY/AgRP neurons (Herzer et al., 2012).

**Figure 2:**
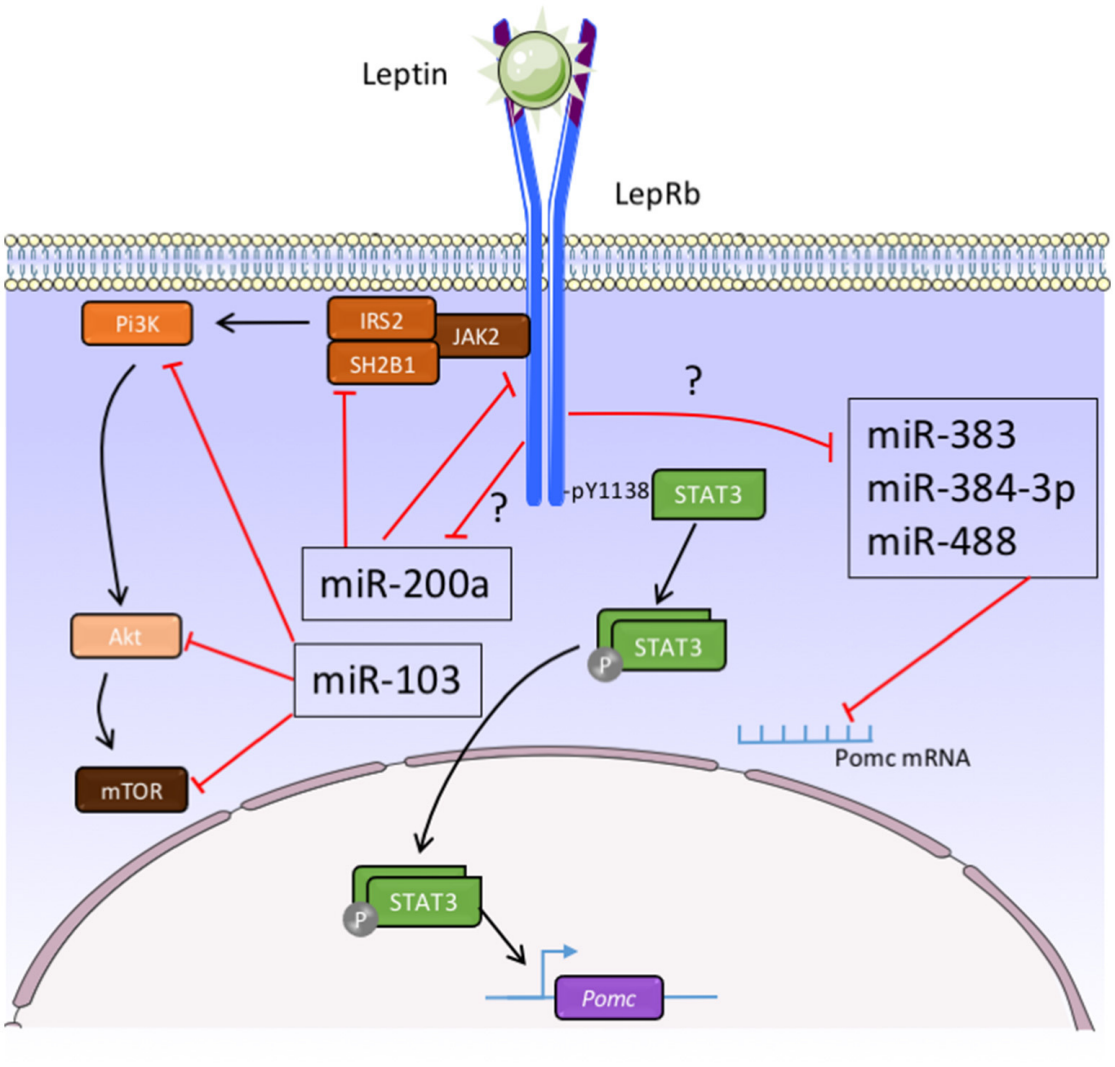
**Function of micro-RNAs in the control of melanocortin neurons activity by leptin**. Akt, **s**erine-threonine protein kinase; IRS-2, insulin receptor substrate 2; JAK2, janus kinase 2; LepRb, leptin receptor type b; mTOR, mammalian target of rapamycin; Pi3K, phosphatidylinositol 3-kinase; POMC, pro-opiomelanocortin; SH2B1, Src homology 2 B adaptor protein 1; STAT3, signal transducer and activator of transcription 3; ?, unexplored pathways.

The functions of hypothalamic miRNAs are highly investigated. In particular, potential impact of leptin on hypothalamic miRNAs expression profile begins to be clarified. Recently, the group of Dr Taouis performed a large-scale expression analysis using Taqman Low Density Arrays methodology to analyse 524 rodent mature miRNAs on the hypothalamus of *ob/ob* mice (Crépin et al., 2014). They showed that the relative expression of only 11 out of 524 miRNAs were significantly modified in the hypothalamus of *ob/ob* mice compared to the control animals (Crépin et al., 2014). They confirmed the over-expression of miR-200a, miR-200b, and miR-429 in *ob*/*ob* mice as compared to control animals by real time PCR (Crépin et al., 2014). Interestingly, the expression of these miRNAs in *ob*/*ob* mice decreased after leptin treatment (Crépin et al., 2014). Importantly, the same group showed that overexpression of mir-200a in *ob/ob* mice can down-regulate Insulin receptor substrate-2 and leptin receptor hypothalamic expression that are involved in the insulin and leptin pathways (Crépin et al., 2014) (Figure 2). In other set of experiments, the group of Dr Taouis demonstrated that the defect in the leptin action in early life supports leptin resistance and disturbs the hypothalamic miRNA expression pattern in adulthood (Benoit et al., 2013). And in particular, daily injection of a pegylated rat leptin antagonist (pRLA) in newborn rats induced a modification of the hypothalamic miRNAs pattern expression at d28 (Benoit et al., 2013). Interestingly, after 1 month of HFD challenge, there is an up-regulation of miR-200a expression in the hypothalamus of pRLA (Benoit et al., 2013). These different observations suggest that miRNAs, and particularly miR-200a, are involved in the effect of leptin and insulin in the hypothalamus (Figure 2). In accordance with these studies, Sangiao-Alvarellos et al. demonstrated the alteration of the hypothalamic expression of a set of miRNAs, including let-7a, mir-9, mir-30e, mir-132, mir-145, mir-200a, and mir-218, after a chronic caloric restriction and a HFD in male rats (Sangiao-Alvarellos et al., 2014). The predicted targets of these miRNAs include different actors of key inflammatory and metabolic pathways, including such as nuclear factor κβ, ILs, phosphatidylinositol 3-kinase (Pi3k)/serine-threonine protein kinase (Akt), insulin receptor, p70S6K, and Janus tyrosine kinase/signal transducer and activator of transcription (Sangiao-Alvarellos et al., 2014). Vinnikov et al. noticed that the injection of mir-103 mimic in the ARC reduced the obese phenotype of mice lacking Dicer in forebrain neurons (Vinnikov et al., 2014). The effect of miR-103 could be associated to Pi3K/Akt/mTOR signaling pathway (Vinnikov et al., 2014) (Figure 2).

Regarding the functions of miRNAs in the POMC neurons, it has been demonstrated that specific deletion of Dicer in POMC-expressing cells leads to obesity and diabetes which is associated with loss of POMC neurons in the ARC (Schneeberger et al., 2012; Greenman et al., 2013). In our group, we identified mir-383, mir-384-3p, and mir-488 that potentially bind the 3-UTR of POMC mRNA (Derghal et al., 2015) (Figure 2). Using *in situ* hybridization, we demonstrated that these three miRNAs are present in the POMC neurons of the ARC (Derghal et al., 2015). In addition, there is an increase of the expression of mir-383, mir-384-3p, and mir-488 in the hypothalamic structures of *ob/ob* and *db/db* mice models (Derghal et al., 2015) (Figure 2). The intraperitoneal and intracerebroventricular injection of leptin decreased the expression of these miRNAs in the hypothalamus of wild type and *ob/ob* mice suggesting a role of leptin in the expression of mir-383, mir-384-3p, and mir-488 (Derghal et al., 2015) (Figure 2). Altogether, these observations strongly suggest that miRNAs are important for the central regulation of energy homeostasis by melanocortin pathway.

## Conclusions and perspectives

As indicated above, a large number of miRNAs are involved in the central regulation of energy homeostasis. We postulate that miRNAs are energy sensors involved in the hypothalamic control of systemic energy balance (Figure 1). Moreover, we cannot exclude a role of the miRNAs in the cortico-limbic system involved in the interaction of organism with the food-providing environment. The complete regulatory network involving miRNAs is largely unknown. However, several studies have identified specific miRNAs that control the expression of the melanocortin pathway genes by leptin (Figure 2). Despite these promising observations, the specific roles of these different miRNAs in the melanocortin pathway neurons activity upon regulation of food intake, energy expenditure, and glucose homeostasis remain largely unknown. Great efforts should also be made to clarify this last point. To address this question, CRISPR/Cas9 as emerging genome editing tool in biology/medicine research could be used. Indeed, CRISPR/Cas9 shows a benefit in the specific control of crossing off-target impact on miRNAs in the same family or with highly similar sequences.

## Author contributions

LM and AD wrote the manuscript. MD and JT helped with manuscript preparation.

### Conflict of interest statement

The authors declare that the research was conducted in the absence of any commercial or financial relationships that could be construed as a potential conflict of interest.
